# Morphological Effects of Natural Products on *Schizosaccharomyces pombe* Measured by Imaging Flow Cytometry

**DOI:** 10.1007/s13659-014-0004-8

**Published:** 2014-02-09

**Authors:** Joel Heisler, Lindsay Elvir, Farah Barnouti, Erica Charles, Tom D. Wolkow, Radha Pyati

**Affiliations:** 1 University of North Florida, Jacksonville, FL USA; 2 University of Colorado at Colorado Springs, Colorado Springs, CO USA

**Keywords:** *Schizosaccharomyces pombe*, Morphology, Natural products, Imaging flow cytometry, Aspect ratio, Fission yeast

## Abstract

Gaining a full understanding of the mechanisms of action of natural products as therapeutic agents includes observing the effects of natural products on cellular morphology, because abnormal cellular morphology is an important aspect of cellular transformations that occur as part of disease states. In this study a set of natural products was examined in search of small molecules that influence the cylindrical morphology of fission yeast *Schizosaccharomyces pombe*. Imaging flow cytometry of large populations of *S. pombe* exposed to natural products captured cell images and revealed changes in mean length and aspect ratio of cells. Several natural products were found to alter *S. pombe*’s morphology relative to control, in terms of elongating cells, shrinking them, or making them more round. These results may facilitate future investigations into methods by which cells establish and maintain specific shapes.

Gaining a full understanding of the mechanisms of action of natural products as therapeutic agents includes observing the effects of natural products on cellular morphology, because abnormal cellular morphology is an important aspect of cellular transformations that occur as part of disease states. In this study a set of natural products was examined in search of small molecules that influence the cylindrical morphology of fission yeast *Schizosaccharomyces pombe*. Imaging flow cytometry of large populations of *S. pombe* exposed to natural products captured cell images and revealed changes in mean length and aspect ratio of cells. Several natural products were found to alter *S. pombe*’s morphology relative to control, in terms of elongating cells, shrinking them, or making them more round. These results may facilitate future investigations into methods by which cells establish and maintain specific shapes.
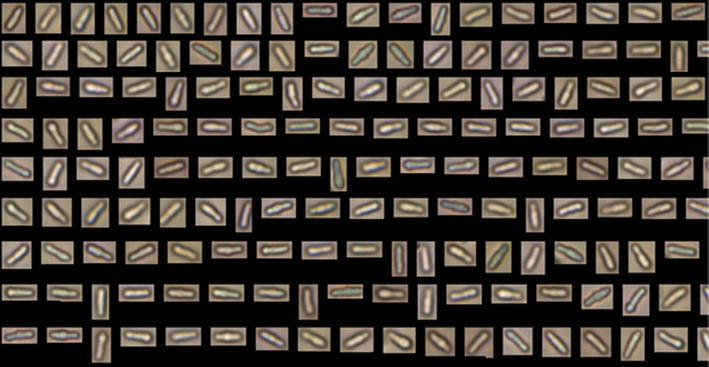

## Introduction

Cell morphology is frequently altered in cancer cells, which are phenotypically categorized based largely upon morphological characteristics. It is generally assumed that genomic mutations in components of signaling networks directly or indirectly result in morphological alterations that allow growth advantages [1]. For example, mutations that allow cells to sample a wider variety of morphological space may permit delamination and metastasis. In support of this view, a genome wide screen in search of genes that regulate cell shape transitions in Drosophila hematocytes led to the identification of mutations in a subset of signaling genes, including the tumor suppressor PTEN, that produce nonheterogeneous populations of cells that switch between rounded and elongated morphologies [2]. Switching back and forth between these shapes may allow cells to sample a large amount of cellular space in search of a shape that permits metastasis. For example, a rounded shape may facilitate passage through the bloodstream while an elongated shape may facilitate migration through harder tissues like bone. The intermediate shapes that arise when these mutant cells switch from round to elongated and vice versa may permit other events of metastasis, such as delamination from an epithelial layer [2].

High-throughput screens have led to the identification of small molecules that specifically alter mammalian morphology and facilitate biomedical discoveries [3, 4]. However, major challenges in isolating morphological effects face those attempting to isolate these chemicals in a high-throughput manner using mammalian cells. For example, such assays require a large population of dividing cells. These populations are typically assembled using cancerous cell lines that already have altered morphology together with genomic mutations capable of masking effects of small molecules. The rampant crosstalk that occurs among mammalian signaling pathways can also mask the effect of small molecules. Furthermore, mammalian cells are wired with many signaling pathways, such as developmental and survival pathways, that influence morphology in a variety of ways. Therefore, small molecules found to alter mammalian morphology may do so indirectly by modulating one of these pathways. In this case it becomes difficult to separate physiological changes that result from morphological alterations as opposed to abnormal cell signaling.

*Schizosaccharomyces pombe* is a model system for investigating pathways that eukaryotic cells use to sample morphological space [5]. Yeast like *S. pombe* also appear to have a reduced level of signaling crosstalk, given that a full yeast protein–protein interaction network contains roughly 37,800–75,500 interactions while the human network is made up of ~154,000–369,000 interactions [6]. Previously, we demonstrated that image flow cytometry (IFC) can be used to accurately capture and quantify the morphological changes that accompany specific *S. pombe* mutations [7]. For example, IFC was used to accurately describe the more spherical appearance of cells that lack *rad24*^+^, a nonessential gene that encodes a 14-3-3 protein [8]. Whereas wildtype cells are cylindrical and have aspect ratios (AR, calculated as width/length) of 0.47, *rad24* mutants are more spherical and found to have elevated ARs (0.58). IFC was also able to accurately capture and quantify the predicted changes in cell length and AR known to accompany treatment following a variety of DNA damaging agents and cytoskeletal toxins. Therefore, IFC is a powerful tool that can be used to accurately describe morphological changes that occur in *S. pombe* as a result of genetic and chemical manipulations.

**Fig. 1:**
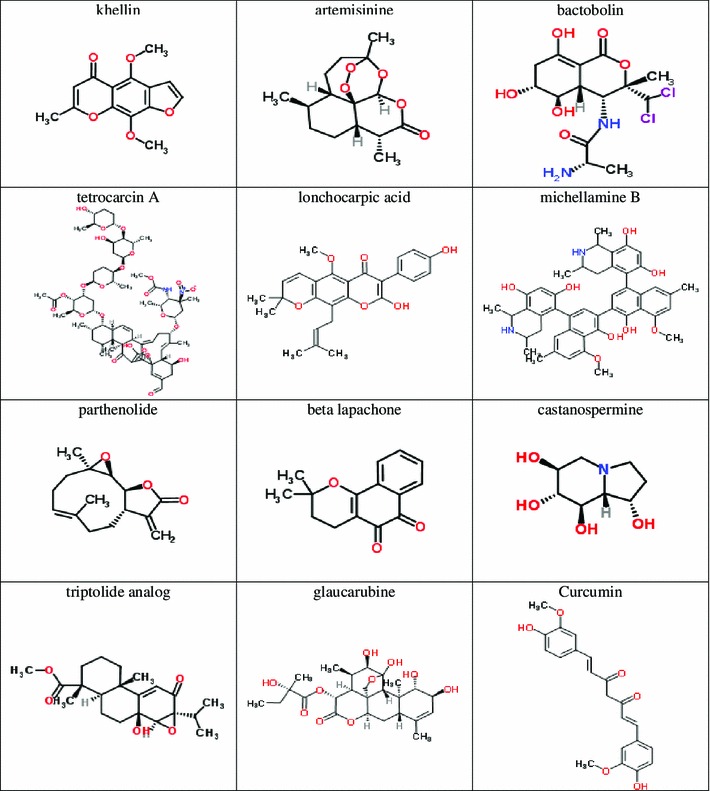
Natural products from NCI Open Chemical Repository Collection Natural Products Set II

Given the power of IFC to sensitively quantify morphological changes resulting from chemical exposure, the United States National Cancer Institute Natural Products Set II was an interesting set of compounds to explore. The set contains many natural products with known medicinal or therapeutic effects. In this work, we selected twelve compounds in the NCI natural products library with well-known medicinal or therapeutic uses for molecules, and we observed their ability to alter length or AR of *S. pombe*. Those twelve compounds appear in Fig. 1.

It is important to note that we are reporting on the morphological changes that occur following exposure to these natural products, not cell population growth as has been reported on previously [9]. Therefore, ours is the first report to address the effects of these compounds on yeast cell shape.

## Results and Discussion

**Fig. 2:**
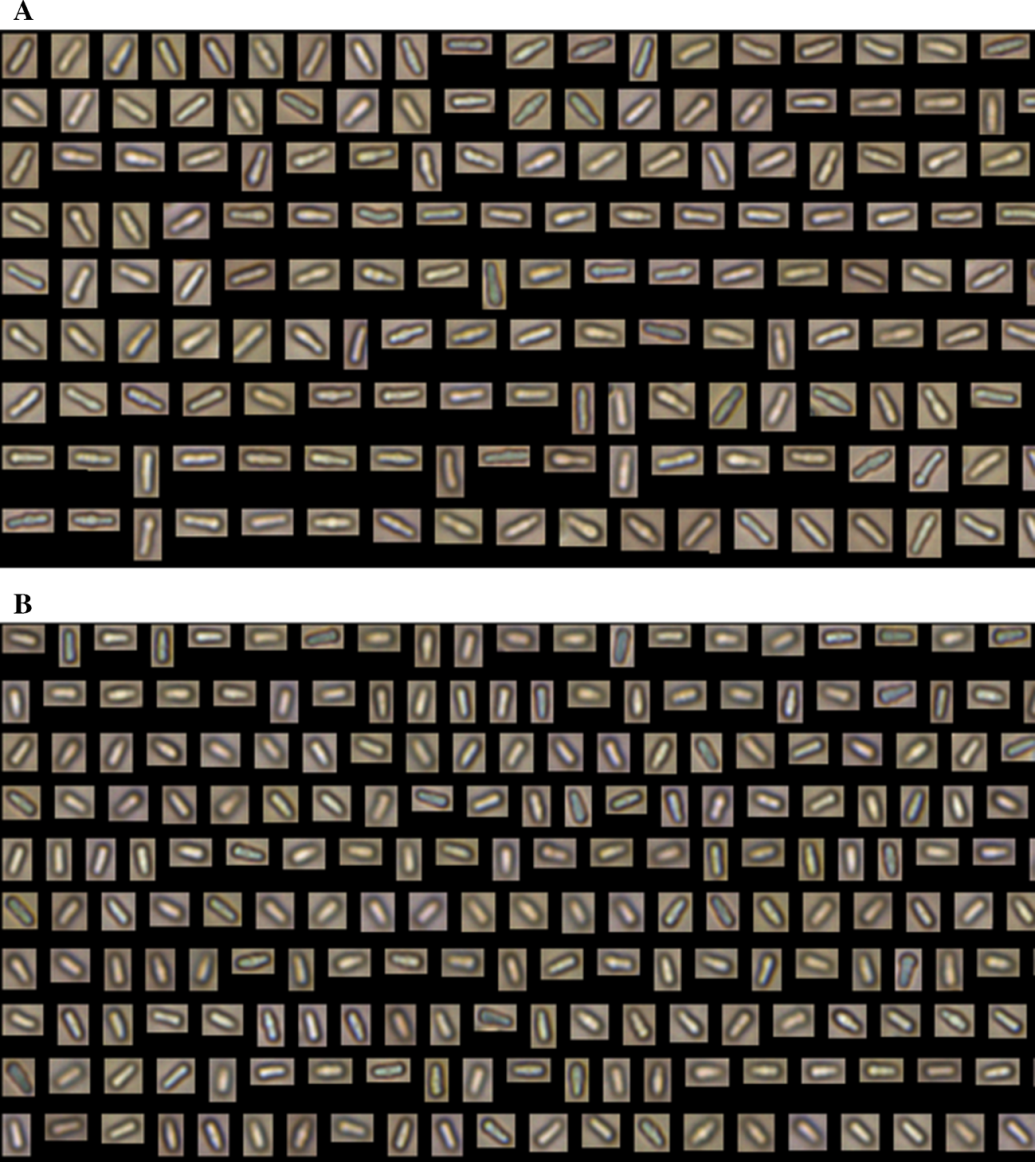
Images collected with FlowCAM IFC. Panels contain most elongated images from each representative sample, for comparison. **A***S. pombe* cells subjected to khellin (NSC#8519) exhibiting with lengths ranging from 10.29 to 11.51 µm represented. **B***S. pombe* cells subjected to no NP (control) with lengths from 9.09 to 9.88 µm

Figure 2 shows two representative examples of image data collected in this study. Cells exposed to khellin are shown in Panel A, and untreated control cells are shown in Panel B. Both panels display the most elongated cells in the data set. The khellin-exposed cells shown range from 10.29 to 11.51 um in length, whereas the control cells shown range from 9.09 to 9.88 µm. This comparison indicates that the longest khellin-exposed cells are longer than the longest control cells. Although this is a comparison of the longest cells in both sets, this analysis is reflected in the fact that the mean length of khellin-exposed cells is 114.7 % of the mean length of control cells.

Table 1 shows cell length and aspect ratio ratioed to control after treatment with each natural product (NP), for each of three trials for each natural product. Standard deviations were calculated from the standard deviations of the NP-exposed value and control value, using a partial derivative formula [10]. An L/Lc less than 100 % indicates that the cells are shorter than the control, and an L/Lc greater than 100 % indicates that cells are longer than the control. Aspect ratio (AR), which is calculated as width divided by length, becomes smaller as cells become more elongated. So an AR/ARc less than 100 % indicates that cells are narrower and more elongated than the control. AR/ARc greater than 100 % indicates that cells are more rounded than the control. As a comparison, and in order to estimate the strength of observed effects, Table 1 also shows cell length and aspect ratio for an established fungicidal drug, phleomycin, which exhibits L/Lc of 157.9 % and AR/ARC of 64.6 %.

**Table 1:** Cell length and aspect ratio relative to control for natural products

Natural product and trial number	L/Lc^a^ (%)	AR/ARc^b^ (%)	Mean L/Lc (%)	Mean AR/ARc (%)
Khellin				
Trial 1	121.7	94.1	114.7 ± 9.2	97.5 ± 8.0
Trial 2	112.6	101.6		
Trial 3	109.7	96.6		
Artemisinine				
Trial 1	101.0	106.8	103.9 ± 3.6	106.0 ± 2.1
Trial 2	106.0	106.5		
Trial 3	104.8	104.7		
Beta-lapachone				
Trial 1	103.7	98.6	102.9 ± 5.3	98.9 ± 3.9
Trial 2	100.4	99.3		
Trial 3	104.7	98.9		
Lonchocarpic acid				
Trial 1	102.7	109.5	101.4 ± 2.8	111.5 ± 2.7
Trial 2	101.5	112.0		
Trial 3	99.9	113.0		
Castanospermine				
Trial 1	99.9	100.3	101.0 ± 4.8	98.9 ± 4.5
Trial 2	100.9	100.3		
Trial 3	102.0	96.3		
Curcumin				
Trial 1	101.4	103.9	100.5 ± 5.0	101.6 ± 4.5
Trial 2	101.9	99.8		
Trial 3	98.2	101.0		
Glaucarubine				
Trial 1	92.6	102.5	98.1 ± 6.6	100.9 ± 4.3
Trial 2	101.2	101.2		
Trial 3	100.5	99.2		
Triptolide				
Trial 1	97.3	101.5	98.0 ± 5.8	99.3 ± 7.5
Trial 2	98.5	96.9		
Trial 3	98.1	99.6		
Parthenolide				
Trial 1	103.4	89.6	98.0 ± 4.9	91.4 ± 2.8
Trial 2	94.6	91.8		
Trial 3	95.9	92.7		
Michellamine B				
Trial 1	94.5	90.3	97.0 ± 2.5	92.9 ± 3.4
Trial 2	98.0	95.1		
Trial 3	98.5	93.3		
Tetrocarcin A				
Trial 1	96.2	99.7	96.7 ± 1.6	99.4 ± 2.7
Trial 2	95.9	100.2		
Trial 3	98.0	98.2		
Bactobolin				
Trial 1	95.7	99.3	92.2 ± 6.3	97.6 ± 2.9
Trial 2	95.8	97.2		
Trial 3	85.0	96.4		
Phleomycin [7]			157.9 ± 17.5	64.6 ± 13.1

^a^L/Lc = mean length of NP-exposed cells divided by mean length of control cells (not exposed to NP), as a percentage. Both NP-exposed and control measurements were performed in triplicate

^b^AR/ARc = mean aspect ratio of NP-exposed cells divided by mean length of control cells (not exposed to NP), as a percentage

Khellin and artemisinine produced the longest *S. pombe* cells, resulting in 114.7 and 103.9 % respective increases in mean length over that of the control cells. Bactobolin and tetrocarcin A produced the shortest cells, 92.2 and 96.7 % respectively, when compared to untreated controls. Lonchocarpic acid and artemisinine increased the AR of cells considerably, yielding 111.5 and 106.0 % of control respectively. Michellamine B and parthenolide treatments significantly reduced the AR of cells, to 92.9, and 91.4 %, respectively. Five NPs showed no measurable effect: beta-lapachone, castanospermine, curcumin, glaucarubine, and triptolide analog.

Each NP is discussed below, in terms of its chemical and species family, any known medicinal properties, other pertinent information about its mechanism of action that yield those properties, and its effects on *S. pombe* cell shape observed in this study. Connections are made between a NP’s effects on *S. pombe* and these other properties wherever possible.

### Khellin

Khellin is a member of the furanochromone family. It is commercially available but was originally used and then isolated from the flowering plant *Ammi visnaga*, native to the Middle East [11]. Khellin is a vasodilator and bronchodilator [12]. It has been implicated, along with its chemical relative visnagin, in the inhibition of nuclear factor κB [13]. It is an anti-inflammatory agent, and its mechanism of action is blocking the calcium channel in cells [11]. In *S. pombe*, khellin has the effect of lengthening cells: L/Lc is considerably larger than 100 %. But AR is relatively unchanged, so width is commensurately increasing along with length. This demonstrates that khellin has enlarged the cell. It may be doing so by affecting the *S. pombe* calcium channel and leading to altered intracellular calcium concentrations are important for cell wall integrity, cytokinesis, and cytoskeletal organization [14]. The mechanical strength of the cell wall may also be affected by intracellular calcium. These effects may weaken the overall cell wall and causing the cell to enlarge, possibly by allowing in higher volumes of water or media into the intracellular space. Another possibility is that Khellin may damage DNA and activate cell cycle checkpoints that lead to the production elongated cells. Or khellin may work by an entirely different mechanism that has yet to be identified.

### Artemisinine

Artemisinine is a sesquiterpene lactone that contains an endoperoxide bridge. It originates from the wormwood plant *Artemisia annua*, is used in traditional Chinese medicine, and is a well-known antimalarial agent. The endoperoxide bridge is essential for its antimalarial activity [15], because it breaks up to form carbon-centered radicals that are destructive to the malaria parasite *Plasmodium falciparum*. This free radical formation requires iron or heme to occur.

In this study, artemisinine produces an AR/ARc greater than 100 % in *S. pombe*, meaning that the cells are rounder than control. This indicates some alteration of the polarity instructions that regulate the cytoskeletal components that coordinate growth along the short and long axes.

### Bactobolin

Bactobolin is a polyketide-peptide produced by a variety of bacterial species including *Pseudomonas* and *Burkholderia thailandensis* [16, 17]. It is a well-characterized antibiotic and antitumor compound. It has been shown to induce apoptosis in melanoma and lymphoma cells [18].

In *S. pombe*, bactobolin is the NP with the smallest L/Lc ratio. This means that the mean length of cells treated with bactobolin is less than those without NP. However, aspect ratio does not change to a similar degree, within experimental error. This NP therefore produces short cells that retain normal length and width proportions. So when both length and width are commensurately decreased, overall growth has decreased. Since the beginning of apoptosis is heralded by cell shrinkage in both upper and lower eukaryotes [19, 20], it is possible that this small cell size results from apoptosis. Clearly, more research is required to address how bactobolin alters *S. pombe* morphology.

### Tetrocarcin A

Tetrocarcin A is an antibiotic isolated from *Micromonospora chalcea*, an actinomycete. It acts as an antibiotic against gram-positive bacteria [21]. It has been found to inhibit anti-apoptotic activity of Bcl-2, a membrane protein responsible for generating anti-apoptosis signals in cells [22].

In *S. pombe*, tetrocarcin A also shows a L/Lc less than 100 %, indicating that overall growth has decreased. It is not clear how this NP leads to the production of small yeast cells, since events downstream of Bcl-2 differ between mammalian cells and *S. pombe* [23].

### Lonchocarpic Acid

Lonchocarpic acid is a 3-aryl-4-hydroxycoumarin. It originates from species of *Derris* sp. and *Lonchocarpus* sp., which are leguminous vines and climbing shrubs. Extracts of these species have been used by Amazon tribes to catch fish, and roots of both species were found to contain rotenone, a well-known insecticide. The production of rotenone became a major industry in the 1930s [24]. Lonchocarpic acid has been used as a plant insecticide [25]. It is an antifeedant to insect larvae and a natural coumarin [26].

Little appears in the scientific literature specifically about lonchocarpic acid’s mechanism of action for either its antifeedant or coumarin properties. As well, effects of lonchocarpic acid on *S. pombe* have no presence in the literature. However, the mechanism of action of rotenone, which bears some structural similarity to lonchocarpic acid, is that rotenone interferes with electron transport.

In *S. pombe*, lonchocarpic acid exhibits an AR/ARc considerably higher than control, indicating that cells are more rounded than control. Cell polarity instructions are therefore compromised. This may be due to generation of free radicals that affect the cytoskeleton, in a mode similar to that of artemisinine. Although lonchocarpic acid may not require heme to operate, its potential interference with electron transport may imply the presence of radicals that affect cell polarity in a similar manner.

### Michellamine B

Michellamine is an alkaloid that originates from the tropical liana plant *Ancistrocladus korupensis*. It is an antiviral agent [27]. It inhibits cell killing by HIV in two pathways: inhibition of both reverse transcriptases as well as processes of cellular fusion. It is considered a leading anti-HIV agent such that efficient routes for its synthesis are being sought and achieved [28]. It has been found that michellamine B also inhibits protein kinase C in the rat brain [29].

Michellamine B yields an AR/ARc lower than 100 %, so the cells are elongated. It seems to shorten the cells slightly as well, although within experimental error: L/Lc is very close to 100 %. This is consistent with the inhibition of protein kinase C described above. *S. pombe* has a family of protein kinase C homologues called Rho1p GTPases [30]. When these are inhibited, pombe cells are unsuccessful at maintaining cell polarity and cell wall integrity [31].

### Parthenolide

Parthenolide is a sesquiterpene lactone that originates from the feverfew plant *Tanacetum parthenium* but is now commercially available [32]. It is known to strongly inhibit nuclear factor κB and have antitumor and anti-inflammatory properties. Its use in cancer inhibition has been patented [32]. It also inhibits DNA methyltransferase 1, which induces global DNA hypomethylation. So parthenolide acts as a DNA damaging agent.

In *S. pombe*, parthenolide shows a strong effect and produces cells with AR/ARc less than 100 %. This means that the aspect ratio is lower than the control, or the cell is rounded. However, parthenolide does not show a change in L/Lc, so length is not changing. This means that the cells are truly more elongated in shape: the same length but less wide. This behavior may be attributable to the DNA damage inflicted by parthenolide.

### Inactive Group

Five of the NCI natural products tested showed no effect on L/Lc or AR/ARc. These five are beta-lapachone, castanospermine, curcumin, glaucarubine, and triptolide analog. Each is briefly discussed below.

### Beta Lapachone

Beta lapachone is an orthonaphthoquinone originating from the South American lapacho tree *Tabebuia avellanadae*. It has also shown anti-inflammatory and antibacterial properties [33]. It has shown a variety of bioactive properties that are believed to be due to the quinoid moiety. It is also known to promote apoptotic cell death by sensitizing tumor cells toward DNA damaging.

### Castanospermine

Castanospermine is an N-linked glycan from the Australian chestnut tree *Castanospermum australe*. It possesses known antiviral properties, and it operates by inhibiting glucosidases required to form the envelope proteins surrounding virus particles [34]. These glucosidases are inhibited by castanospermine and its derivatives, because they operate on N-linked glycans on the viral envelope [35]. This affects morphogenesis of the virus particles. In contrast, this compound has relatively little effect on morphogenesis of *S. pombe*. This may be because its cell wall consists largely of mannoproteins, alpha-glucan, and beta-glucan, not N-linked glycans [36].

### Triptolide Analog

Triptolide is an epoxide, and the triptolide analog used in this study is very similar to triptolide except that it has undergone ring opening in several places. Triptolide is extracted from the Chinese herb *Tripterygium wilfordii* Hook F, called the thunder god vine. It has been used in traditional Chinese medicine, and it is an immunosuppressive agent and an anticancer agent. It has been shown to initiate apoptosis mediated by lysosomes in breast cancer cells [37]. This apoptosis also displays a cell shrinkage behavior in these breast cancer cells. Such effects are not observed in *S. pombe* in this study. This may be because *S. pombe* does not possess lysosomes in the sense that mammalian cells do. But *S. pombe* does contain vacuoles that behave similarly and occupy a similar role [38].

### Glaucarubine

Glaucarubine is a terpenoid compound within the general group of quassinoids. It originates from the paradise tree, or *Simarouba glauca*. Glaucarubine is known to act against amebiasis [39]. A closely related compound, glaucarubinone, has been shown to have a variety of anticancer activities, originating from its inhibition of protein synthesis [40]. It also has anti-inflammatory, insecticidal and antilarval activities [41]. Its anti-inflammatory activities are related to its ability to stabilize lysosomes, which reduces the release of enzymes that do tissue damage [42]. Again, this lysosomal effect would not be present in *S. pombe*, but it could relate to vacuoles in *S. pombe*. At the time of this writing, no results on trials of glaucarubin on pombe are published in the scientific literature.

### Curcumin

Curcumin, or diferuloylmethane, is a diketone. It is derived from the root of the plant Curcuma longa and is found in the spice called turmeric derived from that root. Turmeric has been used as a spice, dye, and folk medical remedy for centuries. It is known to have strong antioxidant properties [43]. It is an inhibitor of the nuclear factor κB pathway [44]. None of these properties have a clear connection to cell morphology changes and in *S. pombe*, none are observed.

## Conclusions

Twelve natural products from the NCI Natural Products Set II were tested against *S. pombe*. Seven were found to have significant effects on either cell length or aspect ratio. More research is required to identify the mechanism of action of each NP and thereby help describe cellular pathways that regulate morphology.

## Experimental Section

### General Experimental Procedures

Two-dimensional fission yeast cell images were collected with a FlowCAM imaging flow cytometer (IFC) with peristaltic pump value of 10, 20× microscopic objective, and 26 particle parameters (Fluid Imaging Technologies, Yarmouth, ME). Optical densities at 595 nm (OD_595_) were recorded on a Genesys 10 uv Scanning UV-Vis Spectrophotometer (Thermo Fisher Scientific, Waltham, MA) with 1″ Plastibrand UV-microcuvettes (Sigma-Aldrich, St. Louis, MO) and a blank of YE5S broth media made from yeast extract (Becton-Dickinson Bacto), dextrose (anhydrous, BDH), adenine hemisulfate dehydrate, (MP Biomedicals, Solon, Ohio), l-histidine free base (MP Biomedicals), l-leucine (MP Biomedicals), and uracil (MP Biomedicals) [45]. Cell cultures were prepared with a VWR Incubator (Sheldon Manufacturing, Cornelius, OR) at 30.5 °C and a 120 rpm rotational shake function for liquid solutions. Centrifugation was performed on a VWR Galaxy 16D Centrifuge (Sheldon Manufacturing, Cornelius, OR) at 5,000 rpm over 2 min durations.

### Natural Products

Khellin (NSC#8519), beta-lapachone (NSC#26326), glaucarubine (NSC#14975), curcumin (NSC#32982), tetrocarcin A (NSC#333856), lonchocarpic acid (NSC#307981), artemisinin (NSC#369397), parthenolide (NSC#157035), michellamine B (NSC#661755), castanospermine (NSC#614552), triptolide analog (NSC#337783), and bactobolin (NSC#325014) were obtained from the NCI Open Chemical Repository Collection Developmental Therapeutics Program (NCI/DTP) Natural Products Set II. The NPs were prepared in DMSO as a stock solution which was later added to triplicate biochemical assay analytes to achieve a NP concentration of 10 μM for the study.

### *Schizosaccharomyces pombe* Acquisition

*S. pombe* wild-type fission yeast with genotype leu1-32, h^−^ ura4-d18 was stored on YE5S agar slant media in a 4 °C refrigerator until use. Reanimation of the yeast was accomplished in YE5S media broth with 24 h incubation at 30.5 °C and a 120 rpm rotational shake.

### Triplicate Biochemical Assay

After initial incubation, OD_595_ was measured using liquid YE5S media as a blank. Following starter culture, transfer volumes and growth times for final samples were carefully controlled in order to obtain OD_595_ values between 0.2 and 0.9, so that log-phase growth and sufficient cell populations could emerge, but plateau-stage growth and the accompanying senescence and exhaustion of media nutrients would not take place. Cell populations were asynchronous. The transfer volume (V_transfer_) was calculated using the OD_595_ obtained from the starter culture and the formula below

$$V_{transfer}=\frac{0.3}{actualOD}\left(\frac{1}{2^{N-1}}\right)*V_{final}\;where\;N=\frac{{\text{t}}_{\text{incub}}}{{\text{t}}_{\text{gen}}}.$$

The transfer volume was then halved or cut into thirds as needed. This V_transfer_ was added into 5 mL of YE5S media and incubated at 30 °C and 120 rpm for a minimum of 12 h. Following this uniform growth period, OD_595_ was measured again. If OD_595_ exceeded 0.3, the sample was diluted with media in order to achieve an OD_595_ of 0.3, to ensure that plateau growth and senescence did not occur during the 6 h period of NP exposure. Each individual NP was then added to a triplicate group of cultures to yield a final NP concentration of 10 µM. Control samples did not have any NP added but underwent the same OD_595_ measurement, dilution, and 3 h growth period, constituting a full growth cycle in *S. pombe*. OD_595_ was measured once more; all final OD_595_ values lay between 0.2 and 0.9. The samples were centrifuged at 5,000 rpm for 2 min, washed in phosphate buffered saline (PBS, 0.2 M Phosphate, 1.5 M NaCl), aspirated, centrifuged again, and resuspended in 70 % cold ethanol to preserve samples. The samples were stored at 4 °C until ready for flow cytometric analysis.

### Imaging Flow Cytometry

Samples in ethanol were resuspended in PBS for analysis using the IFC. Samples underwent two cycles of the following: centrifugation at 5,000 rpm for 2 min, removal of supernatant, dilution in PBS, aspiration, and vortexing to break-up cell clumps. Final samples in PBS were allowed to incubate for 30 min prior to flow cytometric analysis. Eight to ten drops of the sample solution was placed into the opening of the FlowCAM IFC and 50,000 images were collected. A library, consisting of 75 good cell images collected as a skeleton representative of short to long lengths, was made for each set of images corresponding to NP or control samples. FlowCAM software was then instituted to sort by like particle values, resulting in a refined set of predominantly acceptable images. Manual image deletion that was uniform across three individuals was performed until only single cell images remained, with an acceptable final image count at or above 8,000.

### Data Analysis

A table containing 26 parameters for each cell was exported to Microsoft Excel. The two parameters of interest in this work were length and aspect ratio; mean values of these were calculated in Excel. Triplicate trials of each experiment yielded average values and standard deviations for both mean length and aspect ratio.
